# Nanoscale etching characteristics for controlled AFM tomography of amorphous chalcogenide thin films

**DOI:** 10.1186/s42649-026-00147-5

**Published:** 2026-11-03

**Authors:** Siwon Park, Young-Min Kim, Jong-Souk Yeo

**Affiliations:** 1 School of Integrated Technology, College of Computing, Yonsei University, Incheon, 21983 Republic of Korea; 2 BK21 Graduate Program in Intelligent Semiconductor Technology, Yonsei University, Incheon, 21983 Republic of Korea

**Keywords:** Amorphous chalcogenides, Atomic force microscopy, 3D tomography

## Abstract

Amorphous chalcogenide materials are of increasing interest for phase-change memory and threshold-switching device applications. However, the fast-switching behavior in the nanoscale regime is difficult to confirm and characterize in terms of changes in conductivity. Understanding these electrical characteristics requires three-dimensional tomographic imaging capable of resolving structural and compositional changes across the film. Atomic force microscopy (AFM) is a powerful tool for obtaining such 3D tomographic imaging by alternating etching and imaging steps, and it also enables both electrical measurement and biasing during etching, ​which is essential given the volatile nature of Ovonic Threshold Switching (OTS) behavior. However, achieving accurate AFM-based etching for tomography requires a quantitative understanding of the tip-induced wear resistance of the chalcogenide film, since amorphous materials, lacking long-range structural order, make their wear resistance difficult to predict from local bonding configurations and surface-mediated contact mechanics alone. Here, we study a first-order framework for estimating the relative wear resistance of amorphous chalcogenide thin films from Raman spectroscopy and for selecting initial load​ in AFM etching and test it on depth-controlled AFM tomography.

Etched depth as a function of applied load was measured for three amorphous binary films (Ge-Te, Si-Te, and Ge-Se) using AFM with a diamond-coated probe, and the probe state was monitored by force–distance pull-off measurements before and after etching. The load–depth data were analyzed within an Archard–DMT (Derjaguin-Muller-Toporov) framework to obtain a composite wear parameter ($\:{W}_{r}$) that combines the wear coefficient and the effective hardness, allowing relative comparison of the films under nanoscale AFM contact conditions. This parameter follows the order Ge-Te > Si-Te > Ge-Se in both the low- and high-load ranges. To explain this order without independent nanoindentation, we introduce an effective bond enthalpy calculated from the composition and the Raman-resolved bonding configurations, which decreases monotonically with the wear parameter. Applied to an amorphous ternary Si-Ge-Te film, this relationship was used to select an etching load that yielded layer-by-layer tomographic imaging with an etched depth close to the target. These results suggest the Raman-derived effective bond enthalpy as a practical descriptor for selecting initial load​ in AFM-based nanoscale etching of amorphous chalcogenide films.

## Introduction

Amorphous chalcogenide thin films serve a central role in emerging memory technologies, including phase-change memory (PCM) and Ovonic threshold switching (OTS) devices (Wuttig and Yamada 2007; Ovshinsky 1968). The functional performance of these devices depends critically on the structural and mechanical properties of the amorphous phase, which in turn are governed by the composition-dependent bond network formed during deposition (Velea et al. 2017; Zhao et al. 2024; Park et al. 2026).

However, the nanoscale mechanical response of amorphous chalcogenides remains difficult to evaluate quantitatively. Unlike crystalline materials, amorphous materials lack long-range structural order, and their properties are strongly influenced by local bonding configurations, coordination environments and short-range structural heterogeneity. As a result, their mechanical behavior cannot be easily predicted. This difficulty becomes even more pronounced at the nanoscale, where adhesion (Bhaskaran et al. 2010), tip contamination (Liu et al. 2010a, b), and local chemical variation can significantly alter the apparent wear resistance. Therefore, a reliable framework is needed to relate the structural characteristics of amorphous chalcogenide thin films to their nanoscale mechanical and tribological responses.

Atomic force microscopy (AFM) provides a powerful platform for nanoindentation under controlled contact conditions (Buckwell et al. 2019), offering broad observational flexibility across various material systems. (Domke & Radmacher, 1998; Butt et al. 2005) In addition to high-resolution topographic imaging, AFM has been widely used to probe local electrical properties (Kim et al. 2021, 2026; Celano et al. 2013) and to fabricate nanoscale features by controlling the applied load. In particular, AFM-based approaches have been applied to filamentary switching materials, in which sequential nanoscale material removal can be combined with electrical or morphological characterization to reconstruct three-dimensional information about conductive filaments and local device structures (Celano et al. 2014, 2015; Kim et al. 2023).

In this study, we establish a framework for predicting the nanoscale wear resistance of amorphous chalcogenide thin films by linking Raman spectroscopy-derived structural information to AFM-based etching measurements. Rather than treating the wear coefficient and hardness as independently known constants, we introduce an composite wear parameter that incorporates both contributions and enables relative comparison among different amorphous chalcogenide compositions. Using this framework, we demonstrate depth-controlled AFM tomography of amorphous chalcogenide thin films, highlighting its potential as a quantitative approach for nanoscale material characterization and fabrication.

## Materials and methods

**Fig. 1:**
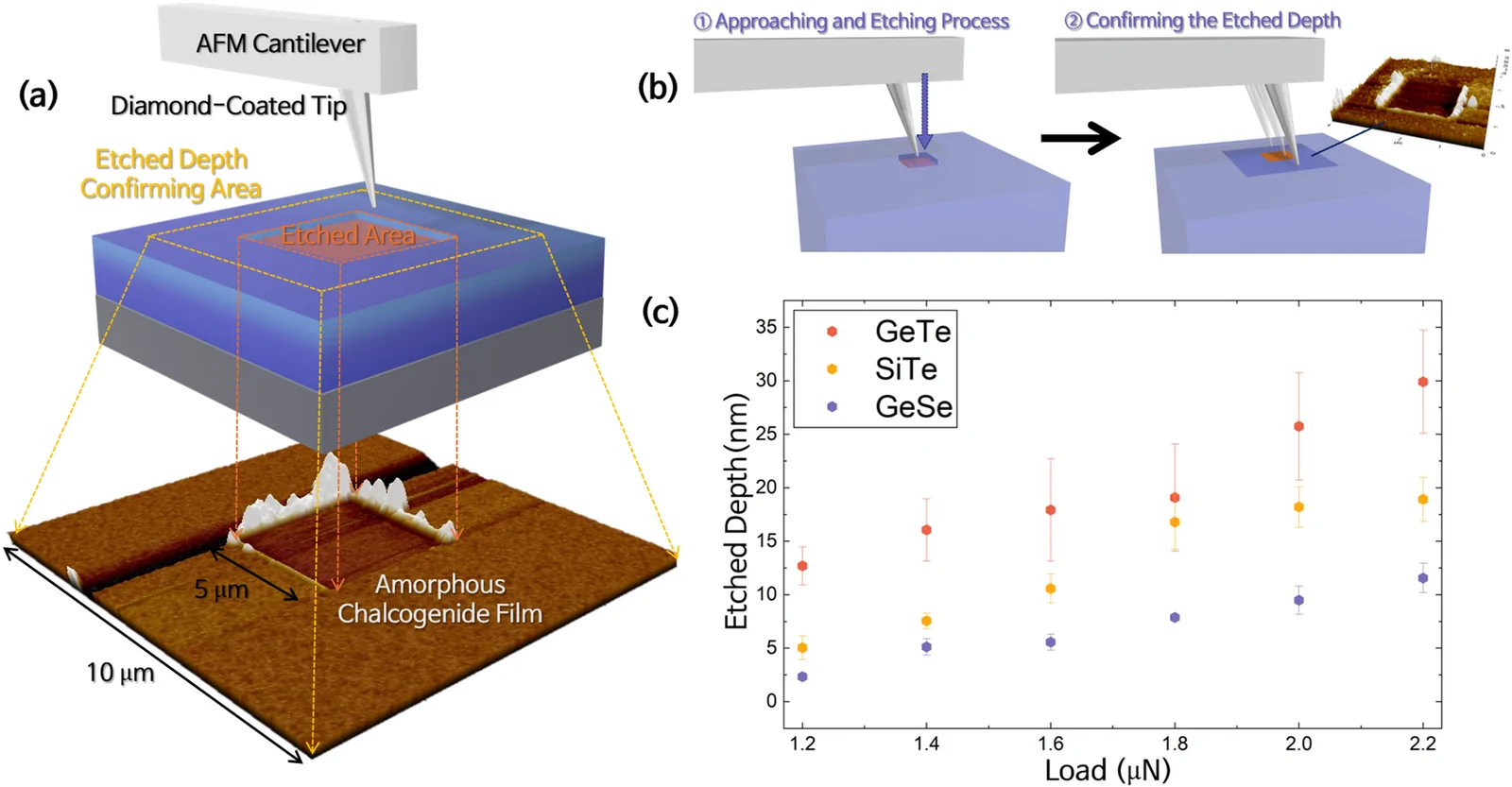
Experimental setup for the etching process of the binary chalcogenide film using Atomic Force Microscopy **a** Etched region (5 × 5 μm², contact mode) and confirming region (10 × 10 μm², non-contact mode) on the film. **b** Schematic of AFM-based etching and depth verification workflow. **c** Etched depth of Ge-Te, Si-Te and Ge-Se thin films

Binary amorphous chalcogenide thin films of Ge_22_-Te_78_, Si_58_-Te_42_ and Ge_77_-Se_23_ were deposited on SiO₂ substrates by RF co-sputtering to a thickness of approximately 50 nm, and their compositions were confirmed by X-ray photoelectron spectroscopy (XPS). Etching was performed in contact mode over a 5 × 5 μm² area (etched region), and the etched morphology and depth were examined in non-contact mode over a 10 × 10 μm² area containing the etched region (confirming region) as shown in Fig. 1(a). Both processes used a diamond-coated conductive probe (CDT-NCHR, Park Systems, nominal tip radius *R* ≈ 100–200 nm and nominal spring constant k ≈ 80 N/m (42–142 N/m). A new probe was used for each film, and all experiments on a given film were performed with the same probe. The CDT-NCHR probe has a polycrystalline diamond-based coating, and diamond and diamond-based films and tips have been reported to have hardness values of approximately 80–120 GPa (Liu et al. 2010a, b; Cicala et al. 2014). The properties of amorphous chalcogenides depend strongly on composition, bonding character, and deposition conditions. Nevertheless, nanoindentation studies of related amorphous chalcogenide systems report hardness values of approximately 1–3 GPa, for example, 1.1–2.4 GPa for As–S and As–Se glasses and thin films (Knotek et al. 2020) and about 2 GPa for amorphous Ge₂Sb₂Te₅ (D’Arrigo et al. 2021). These values are one to two orders of magnitude lower than those of diamond. These values are one to two orders of magnitude lower than those of diamond, which is consistent with the mechanical removal of the present films by the diamond-coated probe.

Before the measurements on each film, the deflection sensitivity of the newly mounted probe was determined from the slope of the contact region of force–distance (F–D) curves on a silicon wafer reference (47 ~ 53 V/µm), and this value was applied to all subsequent measurements together with the nominal spring constant. Etching scans were acquired with 256 × 256 pixels (pixel size and line spacing of 19.5 nm) at a scan rate of 1 Hz, with both trace and retrace in contact. The load was increased from 1200 to 2200 nN in 200 nN increments, with three repeats at each load, each performed at a previously uncontacted location to avoid cumulative damage. All experiments followed the sequence shown in Fig. 1(b): (i) approaching and etching, and (ii) confirming the etched depth. After each etching scan, the same probe was used to reimage the area in non-contact mode (scan rate 0.5 Hz, 256 × 256 pixels, pixel size 39 nm) to measure the etched morphology and depth. The probe state was monitored by F–D curves recorded on unetched areas of the film with the new probe before etching and with the same probe after the 18 etching scans, each condition comprised 16 different positions, and the mean ± standard deviation of the pull-off force was compared in Table 1. After the etching series, the pull-off force decreased by 16% (Ge-Te), 13% (Si-Te) and 26% (Ge-Se) relative to the pre-etch values. Because the pull-off force depends on both the effective tip radius and the adhesion of the probe surface, this decrease indicates a change of the probe state, in which wear or damage of the coating and surface contamination cannot be separated. The pull-off force is therefore used here as an indicator of the magnitude of the probe-state change. For all three films, the mean etched depth increased with increasing load (Fig. 1(c), mean ± standard deviation of *n* = 3 repeats at each load).

**Table 1:** Pull-off force measured on unetched areas with etch probe before and after the etching process (mean ± SD, *n* = 16 positions). The change is given as a percentage of the pre-etch mean

Film	Before etching (nN)	After etching (nN)	Change (%)
Ge-Te	137 ± 11.0	115 ± 24.8	-16
Si-Te	63.7 ± 17.8	55.2 ± 14.4	-13
Ge-Se	76.3 ± 10.4	56.5 ± 11.4	-26

## Results and discussion

### AFM-based Etched Depth Characterization in Binary Chalcogenide Film

**Fig. 2:**
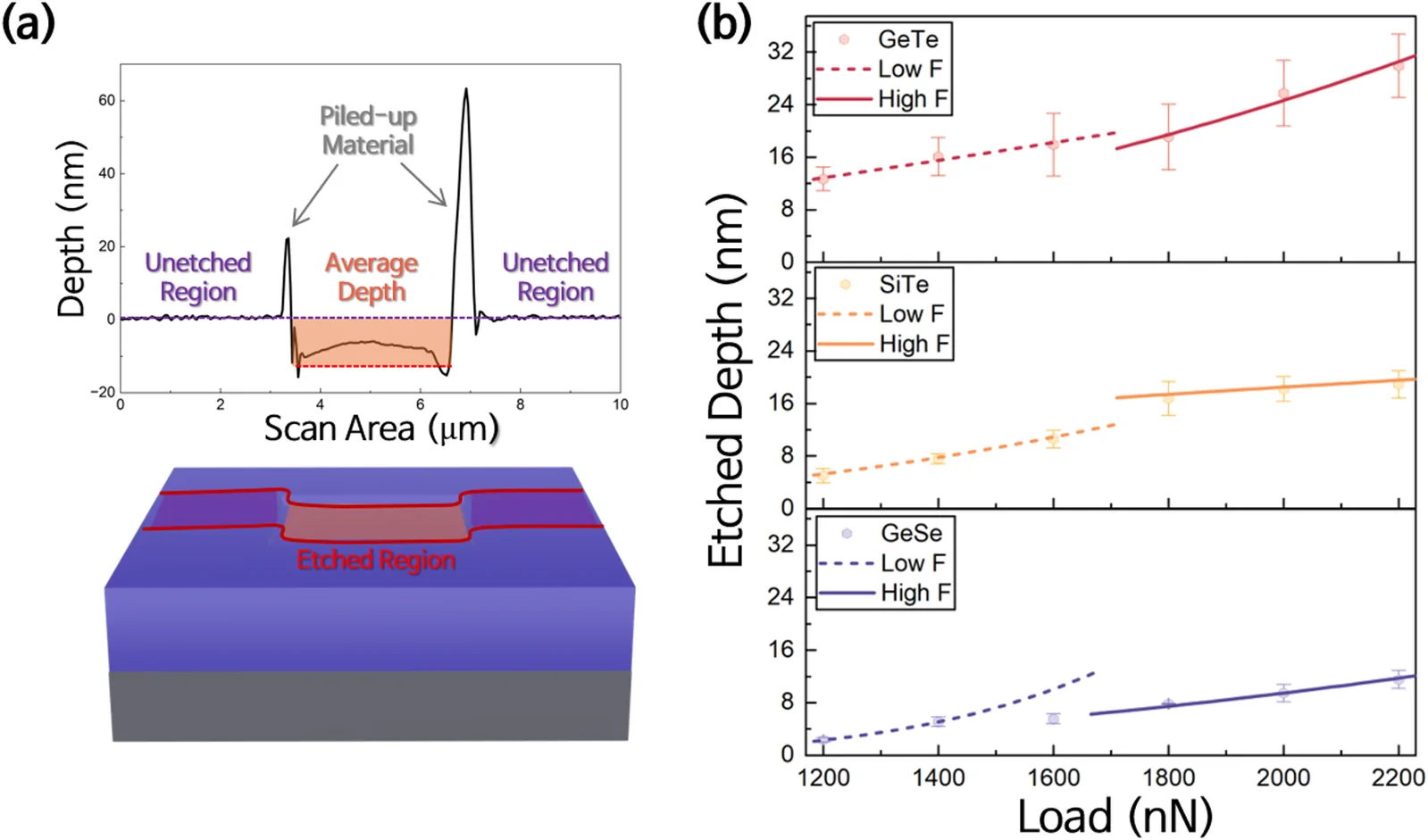
Modeling based on Archard-DMT theory of experimental etched depth **a** Definition of etched depth measured from the average etched depth difference between the unetched region and etched region. **b** Experimental etched depth-load​ curve of Ge-Te (red line), Si-Te (yellow line) and Ge-Se (purple line)

The etched depth(δ) of each chalcogenide film is defined as the mean depth difference between the etched region and the unetched regions of equal height on both sides, excluding the piled-up material along the track edges (Fig. 2(a). To compare the extent of etching among the three materials, the well-known framework (Celano et al. 2018), the Archard sliding wear law (Archard 1953, 1957) combined with Derjaguin-Muller-Toporov (DMT) (Muller et al. 1983) model, was used to account for the spherical tip geometry inherent to AFM-based material etching. The DMT model was adopted because the pull-off forces (adhesion force, $\:{F}_{adh}$) measured by the F-D curves (Table 1) were finite but small compared with the applied loads (about 3–11% of the load), so the adhesion can be treated as an additional force acting outside the contact area. The contact load​ is the $\:{F}_{eff}=F+{F}_{adh}\:$and the contact radius $\:{r}_{c}$ is given by.

1 $$r_c=\left(\frac{3R\left(F+F_{adh}\right)}{4E^*}\right)^{1/3}$$

where R is the tip radius, F is the applied load and the effective modulus E* was taken from the literature (Baloi et al. 2021) In the present geometry, the material volume removed by the AFM tip is given by $\:V=\delta\:\bullet\:2{r}_{c}\bullet\:s$, where 2$\:{r}_{c}$ is the projected contact width and s is the sliding distance. This volume can be combined with Archard’s law $\:V=\frac{K}{H}\bullet\:F\bullet\:s$, in which * K* is the wear coefficient, * H* is the hardness. Since adhesion adds to the load transmitted through the contact, the applied load F is replaced by the effective contact load​ $\:{F}_{eff}$. Equating the two expressions, the sliding distance cancels, and the etched depth can be written in terms of the effective load​ and the contact radius.

2 $$\:\delta\:=\frac{K}{H}\bullet\:\frac{F+{F}_{adh}}{2{r}_{c}},\:\:{W}_{r}=\frac{K}{H}$$

Since the scan size, line density, and number of scans were identical for all films and loads, $\:{W}_{r}$ is an effective parameter defined under these conditions and is used for relative comparison among the films. $\:{W}_{r}$ of each film was obtained by fitting the measured load​–etched depth trend with Eq. (2), separately in a low-load range (1200–1600 nN) and a high-force range (1800–2200 nN), using the same three load points per range for all films (Fig. 2(b), dashed and solid lines). This division was chosen to compare the slopes at low and high loads and does not imply a transition in the wear mechanism.

In both ranges, the three binary films show the same order of $\:{W}_{r}$: Ge-Te > Si-Te > Ge-Se. The $\:{W}_{r}$ values (mean ± standard uncertainty, in 10⁻^10^ m²/N) are 3.63 ± 0.21, 1.91 ± 0.24 and 0.86 ± 0.21 in the low-load range, and 4.94 ± 0.44, 3.62 ± 0.14 and 1.69 ± 0.02 in the high-force range for Ge-Te, Si-Te and Ge-Se, respectively. The differences between the films are larger than the combined standard uncertainties in both ranges. Since $\:{W}_{r}$ is a composite parameter that decreases as the hardness increases, this order indicates that, under identical contact conditions, Ge-Te is relatively the softest and Ge-Se relatively the hardest of the three films. These results show that evaluating the tip–film contact with a classical contact model (DMT) combined with Archard’s law allows the material removal of amorphous chalcogenide films by an AFM tip to be compared among materials. The same order is also expected from the type and number of bonds in each amorphous network, which is examined in the next section using the Raman spectra.

### Derivation of effective bond enthalpy via raman spectroscopy

Amorphous chalcogenides exhibit a wide variety of bond configurations depending on their composition and deposition conditions, and their physical and electrical properties (Lee and Elliott 2022) are known to vary significantly with these structural differences. The mechanical properties of amorphous materials at the nanoscale are known to deviate markedly from macroscopic behavior, since decreasing indentation size leads to an increasing strain gradient relative to the plastic zone, with discontinuous displacement events arising from localized shear transformation (Vo et al. 2019). Consequently, predicting nanoscale hardness trends for amorphous chalcogenides requires a physically grounded predictive descriptor beyond macroscopic hardness data alone to enable reproducible nanofabrication through controlled AFM-based etching.

We evaluate the bond network of each amorphous film by distinguishing homopolar and heteropolar bonding configurations from their short-range ordering. The associated standard bond enthalpies, calculated using Pauling’s rule as reported in the Lankhorst model (Lankhorst 2002), are used to estimate an effective bond enthalpy ($\:{BE}_{\mathrm{e}\mathrm{f}\mathrm{f}}$) for each film, which is then used to compare and predict nanoscale composite wear parameters across the three materials. The calculated values of major bonding are listed in Table 2.

**Table 2:** Major bond enthalpies in three binary materials calculated by Pauling’s relation

Bonding	Te-Te	Ge-Ge	Se-Se	Ge-Te	Si-Te	Ge-Se
H_AB_ (kJ/mol)	197	186	227	192	215	235

**Fig. 3:**
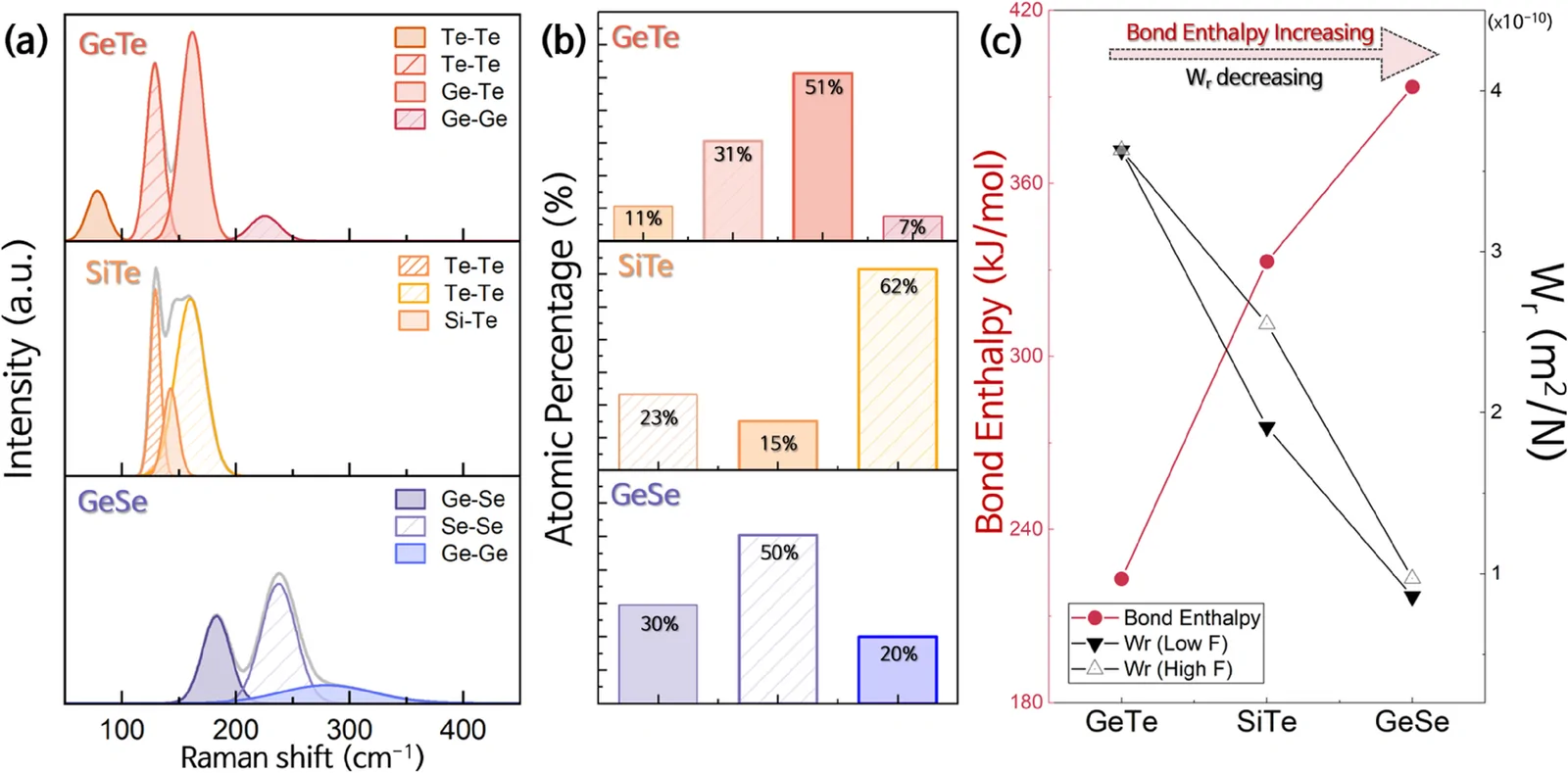
Derivation of effective bond enthalpy via Raman as a descriptor of composite wear parameter (W_r_) **a** Raman spectrum with deconvoluted peaks and **b** Normalized bond fraction of Ge-Te, Si-Te and Ge-Se **c** Correlation between model-derived composite wear parameters and effective bond enthalpy across the materials

The dominant bonding configurations of each film were identified by Raman spectroscopy, and the normalized spectra for the binary Ge-Te, Si-Te, and Ge-Se thin films are shown in Fig. 3(a), with details in Table 3. For the Ge-Te film, three main structural motifs are identified: bending mode of Te–Te chains, Ge–Te stretching modes corresponding to both corner-sharing and edge-sharing tetrahedral units, and Ge–Ge homopolar bonds (Zhao et al., 2025). The Si-Te film shows also Te–Te chain vibrations and Si–Te stretching modes (Lee et al., 2024). The Ge-Se film yields three distinct contributions: Ge–Se bonds (Boolchand et al., 2007), intrachain stretching of Se–Se bonds (Pinto et al., 2012), and broadened Ge–Ge bonds, which is shown in Ge-rich compositions (Ali et al., 2022).

**Table 3:** Deconvoluted Raman peak positions and normalized areas of Ge-Te, Si-Te and Ge-Se films

System	Bonding Type	1/$\:\boldsymbol{\lambda\:}$(cm^− 1^)	Area (%)
Ge-Te	Te-Te Te-Te Ge-Te Ge-Ge	80 cm^− 1^ 128 cm^− 1^ 162 cm^− 1^ 226 cm^− 1^	11% 31% 51% 7%
Si-Te	Te-Te Te-Te Si-Te	130 cm^− 1^ 143 cm^− 1^ 160 cm^− 1^	23% 15% 62%
Ge-Se	Ge-Se Se-Se Ge-Ge	183 cm^− 1^ 238 cm^− 1^ 280 cm^− 1^	30% 50% 20%

As shown by the normalized area from the Raman spectra (Fig. 3(b)), there is a slight discrepancy between the nominal compositional ratio and the actual bonding ratio constituting the thin film. The number of bonds per atom is determined by the elemental composition and an atom α with coordination number $\:{x}_{\alpha\:}$ forms $\:{r}_{\alpha\:}$ bonds, and each bond is shared by two atoms, so that $\:\frac{⟨r⟩comp}{2}$ bonds per atom are present. The Raman area fractions $\:{f}_{k}\:$(Table 3, normalized so that ∑ $\:{f}_{k}$ = 1) then specify the fraction of these bonds belonging to each bond type k. Using the area fraction obtained above as a weighting factor, the effective bond enthalpy ($\:B{E}_{eff}$) of the thin film was calculated by substituting the previously determined bond enthalpy values (Table 2) of each bond into the following equation:

$$\:B{E}_{eff}=\frac{{〈r〉}_{comp}}{2}\sum_f\:{f}_{k}{\bullet\:H}_{k}$$

where $\:{f}_{k}$ is the normalized Raman fraction (Fig. 3(b), Table 3) of the * k*-th bond species and $\:{H}_{k}$ is its bond enthalpy. $\:{〈r〉}_{comp}={\sum\:}_{\alpha\:}{x}_{\alpha\:}{r}_{\alpha\:}$ is the composition-weighted mean coordination number. $\:{BE}_{\mathrm{e}\mathrm{f}\mathrm{f}}$ is thus the bond enthalpy per mole of atoms, the number of bonds per atom times the mean enthalpy per bond. Because the Raman area reflects the scattering cross-section of each mode as well as the bond concentration, $\:{f}_{k}\:$is used as an approximate measure of the bond fraction, and $\:{BE}_{\mathrm{e}\mathrm{f}\mathrm{f}}$ serves as a descriptor for ranking the films rather than as an absolute bond enthalpy.

The resulting $\:{BE}_{\mathrm{e}\mathrm{f}\mathrm{f}}$ values are 223, 333, and 394 kJ mol⁻¹ for GeTe, SiTe, and GeSe, respectively, and are indicated by red markers in Fig. 3(c). This monotonically increasing sequence (Ge-Te < Si-Te < Ge-Se) is in good agreement with the decreasing trend in composite wear parameter $\:{W}_{r}\:$observed across both the low-load and high-load ranges, indicating that films with a weaker average bond network are more sensitive to nanoscale etching. These results suggest that$\:\:{BE}_{\mathrm{e}\mathrm{f}\mathrm{f}}$ derived from Raman spectral analysis can serve as a practical and physically interpretable descriptor for ranking relative composite wear parameters among amorphous chalcogenide thin films.

### 3D Tomography of amorphous chalcogenide film

To validate the predictive capability of the $\:{BE}_{\mathrm{e}\mathrm{f}\mathrm{f}}$–$\:{W}_{r}$ framework beyond the binary systems used to construct it, we applied the model to an amorphous ternary Si_13_-Ge_07_-Te_80_ thin film and used the predicted $\:{W}_{r}$ to guide layer-by-layer tomographic imaging.

**Fig. 4:**
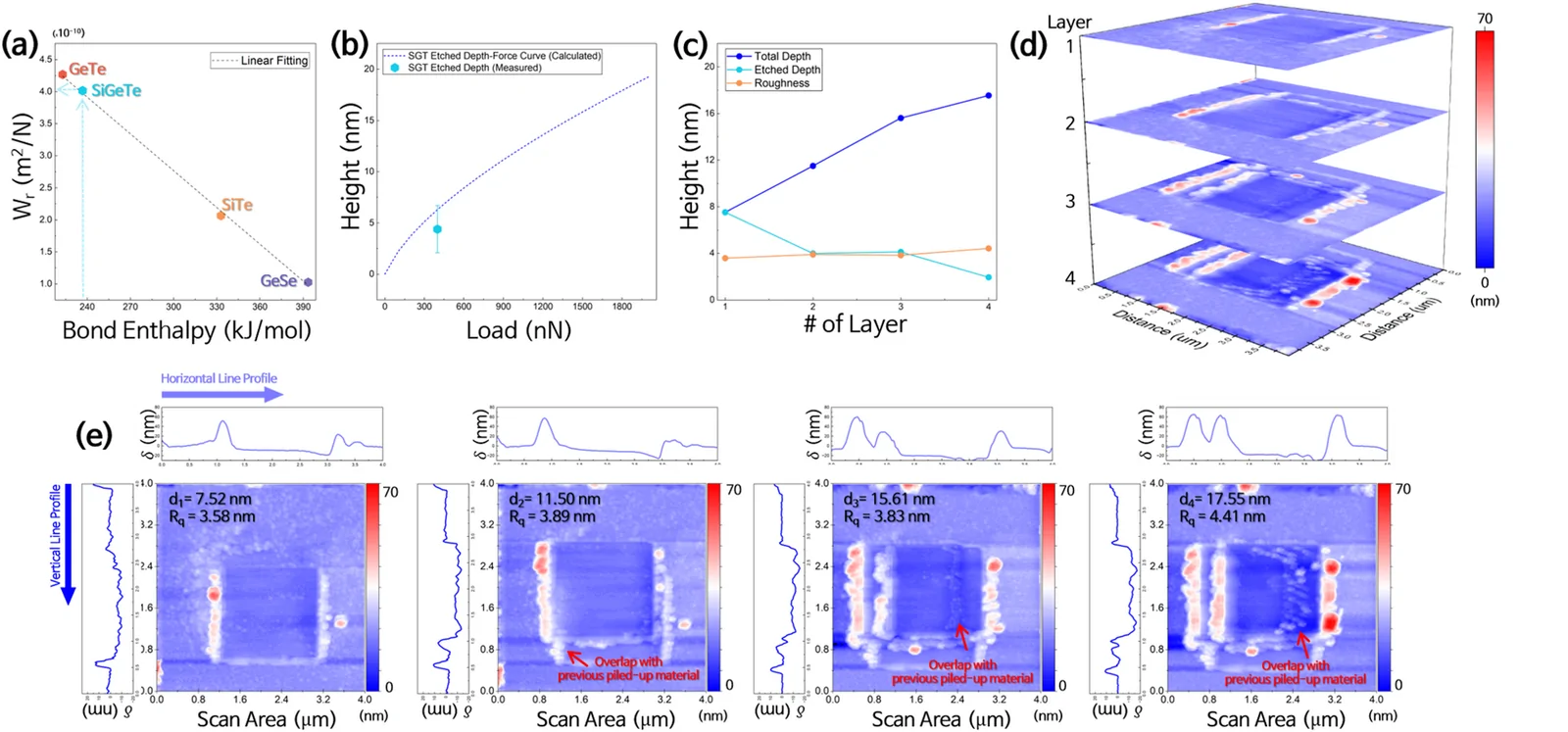
3D Tomography of amorphous chalcogenide Si-Ge-Te film **a** Linear correlation between effective bond enthalpy and composite wear parameter in binary chalcogenide materials and extracted Wr of Si-Ge-Te using its effective bond enthalpy. **b** Calculated etched depth-load​ curve of Si-Ge-Te and experimental etched depth under 350 nN. **c** Etched depth and Roughness for each layer **d** 3D tomography image of Si-Ge-Te **e** Line profile and layer images for each layer

The effective bond enthalpy of the Si-Ge-Te film was first calculated from its Raman spectrum from the literature (Park et al. 2026) using the same intensity-weighted averaging procedure described above. The resulting $\:{BE}_{\mathrm{e}\mathrm{f}\mathrm{f}}$ value was then inserted into the linear fit of the $\:{BE}_{\mathrm{e}\mathrm{f}\mathrm{f}}$–$\:{W}_{r}$ relationship established from the three binary films (Fig. 4(a), yielding a predicted $\:{W}_{r}\:$of 4.01 × 10⁻^10^ m² N⁻¹. Because $\:{W}_{r}=K/H$ encapsulates both the wear coefficient and the effective hardness of the film as a single composite parameter without requiring their independent determination, this prediction directly provides the slope of the etched depth versus applied load curve for Si-Ge-Te (Fig. 4(b), which is assumed to pass through the origin consistent with the adhesion force of Si-Ge-Te measured to be 185 nN from the F-D curve.

Based on this model, an applied load of 350 nN was selected to target an etched depth of approximately 5 nm per layer. Etching was performed by contact-mode scanning over a 2.5 μm × 2.5 μm area at the target load, repeated for four successive layers. Each etching process was followed by a non-contact imaging scan to confirm the etched depth and roughness of each layer (Fig. 4(c)). The depth-resolved positional images are shown in Fig. 4(d), and the per-layer images together with horizontal and vertical line profiles are provided in Fig. 4(e).

Plastic deformation was confirmed in every layer by the presence of piled-up material along the horizontal scan direction, accumulating preferentially at the groove sidewalls and progressively redistributing across the scanned area with increasing etched depth. To make this piled-up material directly visible, a small lateral offset was applied between successive etching passes, exposing the accumulated material at the perimeter of each layer.

Across the four layers, the etched depth was 7.5, 4.0, 4.1 and 1.9 nm (mean ± SD = 4.4 ± 2.3 nm, *n* = 4 layers; 95% confidence interval 0.7–8.1 nm), which is 12.3% below the modelled target of 5 nm. Although the mean value agrees reasonably well with the model, the inter-layer variability is substantial, and surface roughness increased progressively with etched depth. Because the layers were etched successively at the same position with the same probe, they are not ​independent replicates, we therefore regard this result as a first-pass validation of the load selection rather than an accurate prediction.

Two factors likely account for the deviation of the mean from the target. First, the $\:{BE}_{\mathrm{e}\mathrm{f}\mathrm{f}}$–$\:{W}_{r}$ relationship was constructed from binary chalcogenide films, and the linear fit necessarily assumes that the composite parameter $\:{W}_{r}=K/H\:$scales with $\:{BE}_{\mathrm{e}\mathrm{f}\mathrm{f}}$ in the same trend for ternary compositions. In Si-Ge-Te, the additional element introduces cross-bonding configurations, such as Si–Te and Ge–Si interactions, that are absent in the binary training set and may shift both * K* and * H* in ways not captured by the single-variable $\:{BE}_{\mathrm{e}\mathrm{f}\mathrm{f}}$ descriptor. Second, $\:{W}_{r}$ was determined from loads of 1200–2200 nN, whereas the tomography was deliberately conducted at a lower load (350 nN) to achieve the shallow depth required for depth-resolved imaging. Extrapolating $\:{W}_{r}$ to this load, together with the assumed adhesion force, introduces an uncertainty that partially accounts for the observed discrepancy.

The model predicts the same depth for every layer, whereas the measured depth decreased with layer number. The surface images after each step (Fig. 4(e)) suggest that the pile-up formed during the repeated etching of the same area contributes to this decrease. The cumulative depths in Fig. 4(e) were 7.52, 11.50, 15.61, and 17.55 nm, and the depth of each layer is the difference between two consecutive values. Because the same area was etched in every step, the pile-up at the edges of the etched region was formed at the same positions, and the pile-up was largest after the last step. In each following step, the probe collides with this accumulated material, which can reduce the etching efficiency, and this material can also affect the etched region. This reduces the net depth of the later layers, in agreement with the increase of the surface roughness (R_q_) of the etched region from 3.58 to 4.41 nm. The pile-up can be reduced by decreasing the size of the etched region step by step, which should be tested in future work.

Nevertheless, the reasonable agreement between the predicted and measured mean etched depths suggests that the $\:{BE}_{\mathrm{e}\mathrm{f}\mathrm{f}}$-based $\:{W}_{r}$ model can serve as a practical first-pass estimator for selecting appropriate load conditions when performing AFM-based nanoscale etching of amorphous chalcogenide thin films. To our knowledge, this represents the first use of a compositional bond-enthalpy descriptor to predict the $\:{W}_{r}=\frac{K}{H}$ composite wear parameter and thereby guide load selection for nanoscale etching of amorphous chalcogenides across varied compositions, and it lays the groundwork for systematic extension to a broader range of ternary and quaternary systems.

## Conclusions

This work presents a Raman spectroscopy-guided, first-order framework for estimating the relative wear resistance of amorphous chalcogenide thin films and for selecting initial load​ for AFM-based etching with a multi-function diamond-coated probe. Combining the Archard law with a DMT contact model that uses the measured pull-off force, the load dependence of the etched depth of Ge-Te, Si-Te and Ge-Se films was described by a composite wear parameter $\:{W}_{r}=\frac{K}{H}$, which follows the order Ge-Te > Si-Te > Ge-Se in both the low- and high-load ranges. This order decreases monotonically with the effective bond enthalpy $\:\left({BE}_{\mathrm{e}\mathrm{f}\mathrm{f}}\right)$calculated from Raman spectra, which therefore offers a practical, non-destructive descriptor for choosing to start etching conditions without nanoindentation. A test on an amorphous Si-Ge-Te film gave a mean etched depth 12.3% below the 5 nm target, which we regard as a first-pass result.

The results are relative and probe specific. The pull-off force of each probe decreased during etching, the loads are nominal values, and the ternary test was limited to one film with four non-independent layers. Future work should include replicate probes with direct probe observation, monitoring of the probe state during etching, and a larger calibration set including additional ternary and quaternary compositions.

## Data Availability

The data that support the findings of this study are available from the corresponding author upon reasonable request.

## Abbreviations

AFM: Atomic force microscopy
BE_eff_: Effective bond enthalpy from raman spectra

## References

[CR33] A. Ali, S.M. Ansari, B. Ehab, B. Mohammad, D.H. Anjum, H.M. Aldosari, Effect of vacuum annealing on structural and electrical properties of germanium telluride thin films. Mater. Res. Bull. **146**, 111575 (2022)

[CR23] J. Archard, Contact and rubbing of flat surfaces. J. Appl. Phys. **24**(8), 981–988 (1953)

[CR22] J. Archard, (1957). Elastic deformation and the laws of friction. Proceedings of the royal society of London. Series A. Mathematical and physical sciences, 243(1233), 190–205

[CR25] M. Baloi, D. Wamwangi, B. Mathe, R. Erasmus, D. Billing, M. Madhuku, P. Sechogela, (2021). Elastic properties and lattice thermal conductivity of amorphous Ge2Sb2Te5 and GeTe thin films. J. Appl. Phys., **129**(13),135102 10.1063/5.0041242

[CR6] H. Bhaskaran, B. Gotsmann, A. Sebastian, U. Drechsler, M.A. Lantz, M. Despont, P. Jaroenapibal, R.W. Carpick, Y. Chen, K. Sridharan, Ultralow nanoscale wear through atom-by-atom attrition in silicon-containing diamond-like carbon. Nat. Nanotechnol. **5**(3), 181–185 (2010) 10.1038/nnano.2010.3 20118919

[CR31] P. Boolchand, M. Jin, D. Novita, S. Chakravarty, Raman scattering as a probe of intermediate phases in glassy networks. J. Raman Spectroscopy: Int. J. Original Work all Aspects Raman Spectrosc. Including High. Order Processes also Brillouin Rayleigh Scattering. **38**(6), 660–672 (2007)

[CR8] M. Buckwell, W.H. Ng, S. Hudziak, A. Mehonic, M. Lanza, A.J. Kenyon, Improving the consistency of nanoscale etching for atomic force microscopy tomography applications. Front. Mater. **6**, 203 (2019)

[CR10] H.-J. Butt, B. Cappella, M. Kappl, Force measurements with the atomic force microscope: Technique, interpretation and applications. Surf. Sci. Rep. **59**(1–6), 1–152 (2005)

[CR12] U. Celano, L. Goux, K. Opsomer, A. Belmonte, M. Iapichino, C. Detavernier, M. Jurczak, W. Vandervorst, Switching mechanism and reverse engineering of low-power Cu-based resistive switching devices. Nanoscale. **5**(22), 11187–11192 (2013). 10.1039/c3nr03579d 24080868

[CR14] U. Celano, L. Goux, A. Belmonte, K. Opsomer, A. Franquet, A. Schulze, C. Detavernier, O. Richard, H. Bender, M. Jurczak, W. Vandervorst, Three-dimensional observation of the conductive filament in nanoscaled resistive memory devices. Nano Lett. **14**(5), 2401–2406 (2014). 10.1021/nl500049g 24720425

[CR15] U. Celano, L. Goux, R. Degraeve, A. Fantini, O. Richard, H. Bender, M. Jurczak, W. Vandervorst, Imaging the Three-Dimensional Conductive Channel in Filamentary-Based Oxide Resistive Switching Memory. Nano Lett. **15**(12), 7970–7975 (2015). 10.1021/acs.nanolett.5b03078 26523952

[CR21] U. Celano, F.-C. Hsia, D. Vanhaeren, K. Paredis, T.E. Nordling, J.G. Buijnsters, T. Hantschel, W. Vandervorst, Mesoscopic physical removal of material using sliding nano-diamond contacts. Sci. Rep. **8**(1), 2994 (2018) 10.1038/s41598-018-21171-w PMC5813091 29445103

[CR18] G. Cicala, V. Magaletti, G. Senesi, G. Carbone, D. Altamura, C. Giannini, R. Bartali, Superior hardness and Young’s modulus of low temperature nanocrystalline diamond coatings. Mater. Chem. Phys. **144**(3), 505–511 (2014)

[CR20] G. D’Arrigo, M. Scuderi, A. Mio, G. Favarò, M. Conte, A. Sciuto, M. Buscema, G. Li-Destri, E. Carria, D. Mello, Mechanical characterization and properties of continuous wave laser irradiated Ge2Sb2Te5 stripes. Mater. Design. **202**, 109545 (2021)

[CR9] J. Domke ,M. Radmacher, Measuring the elastic properties of thin polymer films with the atomic force microscope. Langmuir. **14**(12), 3320–3325 (1998)

[CR11] Y.M. Kim, J. Lee, D.J. Jeon, S.E. Oh, J.S. Yeo, Advanced atomic force microscopy-based techniques for nanoscale characterization of switching devices for emerging neuromorphic applications. Appl. Microsc. **51**(1), 7 (2021). 10.1186/s42649-021-00056-9 PMC8155164 34037869

[CR16] J. Kim, W. Kim, J. Kim, H. Sohn, Locally formed conductive filaments in an amorphous Ga2Te3 ovonic threshold switching device. AIP Adv. **13**(3) (2023). 10.1063/5.0140715

[CR13] Y.M. Kim, S.B. Lee, S. Park, J.S. Yeo, (2026). Nanoscopic Observation of Structural Changes After First-Firing Behavior in W/GeTe/W Ovonic Threshold Switches Using AFM Tip–Based Local Biasing. Small Methods, e70793 10.1002/smtd.70793 PMC13555642 42317074

[CR19] P. Knotek, P. Kutálek, E. Černošková, M. Vlček, L. Tichý, The density, nanohardness and some optical properties of As–S and As–Se chalcogenide bulk glasses and thin films. RSC Adv. **10**(70), 42744–42753 (2020) 10.1039/d0ra08939g PMC9057988 35514909

[CR28] M. Lankhorst, Modelling glass transition temperatures of chalcogenide glasses. Applied to phase-change optical recording materials. J. Non-cryst. Solids. **297**(2–3), 210–219 (2002). 10.1016/S0022-3093(01)01034-1

[CR26] T. Lee, S. Elliott, Hypervalency in amorphous chalcogenides. Nat. Commun. **13**(1), 1458 (2022) 10.1038/s41467-022-29054-5 PMC8933559 35304462

[CR30] S.-B. Lee, C. Park, S.-H. Park, Y. Lee, J.-S. Yeo, Effect of nitrogen doping on characteristics of SiTe Ovonic threshold switch for selectors. Mater. Sci. Semiconduct. Process. **178** (2024). 10.1016/j.mssp.2024.108470

[CR7] J. Liu, J.K. Notbohm, R.W. Carpick, K.T. Turner, Method for characterizing nanoscale wear of atomic force microscope tips. Acs Nano. **4**(7), 3763–3772 (2010a) 10.1021/nn100246g 20575565

[CR17] J. Liu, D. Grierson, N. Moldovan, J. Notbohm, S. Li, P. Jaroenapibal, S. O’Connor, A. Sumant, N. Neelakantan, J. Carlisle, Preventing nanoscale wear of atomic force microscopy tips through the use of monolithic ultrananocrystalline diamond probes. Small. **6**(10), 1140–1149 (2010b) 10.1002/smll.200901673 20486220

[CR24] V. Muller, B. Derjaguin, Y.P. Toporov, On two methods of calculation of the force of sticking of an elastic sphere to a rigid plane. Colloids Surf. **7**(3), 251–259 (1983)

[CR2] S.R. Ovshinsky, Reversible electrical switching phenomena in disordered structures. Phys. Rev. Lett. **21**(20), 1450 (1968)

[CR5] S. Park, C. Park, S.-B. Lee, Y.-M. Kim, S. Kim, J.-S. Yeo, (2026). Scalable and sustainable N-Si-Ge-Te Ovonic threshold switching devices for energy-efficient artificial neuron applications. ACS Appl. Electron. Mater. 8(14):6121–6130. 10.1021/acsaelm.6c01010

[CR32] A.H. Pinto, E.R. Leite, E. Longo, E.R. de Camargo, Crystallization at room temperature from amorphous to trigonal selenium as a byproduct of the synthesis of water dispersible zinc selenide. Mater. Lett. **87**, 62–65 (2012)

[CR3] A. Velea, K. Opsomer, W. Devulder, J. Dumortier, J. Fan, C. Detavernier, M. Jurczak, B. Govoreanu, Te-based chalcogenide materials for selector applications. Sci. Rep. **7**(1), 8103 (2017). 10.1038/s41598-017-08251-z PMC5556072 28808294

[CR27] T. Vo, B. Reeder, A. Damone, P. Newell, Effect of domain size, boundary, and loading conditions on mechanical properties of amorphous silica: A reactive molecular dynamics study. Nanomaterials. **10**(1), 54 (2019) 10.3390/nano10010054 PMC7022248 31881644

[CR1] M. Wuttig, N. Yamada, Phase-change materials for rewriteable data storage. Nat. Mater. **6**(11), 824–832 (2007) 10.1038/nmat2009 17972937

[CR4] Z. Zhao, S. Clima, D. Garbin, R. Degraeve, G. Pourtois, Z. Song, M. Zhu, Chalcogenide ovonic threshold switching selector. Nano-Micro Lett. **16**(1), 81 (2024). 10.1007/s40820-023-01289-x PMC10784450 38206440

[CR29] Z. Zhao, M. Zhang, Q. Yang, T. Gotoh, Q. Ge, N. Shi, Y. Sun, J. Zhao, Y. Sui, R. Jiang, Volatile to Non-Volatile Switching Transition in Chalcogenides. Adv. Funct. Mater. 2423940 (2025). 10.1002/adfm.202423940

